# P-1085. An Opportunity for Stewardship: Idle Peripheral Venous Catheters

**DOI:** 10.1093/ofid/ofaf695.1280

**Published:** 2026-01-11

**Authors:** Heather Young, Himgauri Nikrad, Rosine Angbanzan, Sarah Gardiner, Kelly Medero

**Affiliations:** Denver Health, Denver, CO; Denver Health, Denver, CO; Denver Health, Denver, CO; Denver Health, Denver, CO; Denver Health, Denver, CO

## Abstract

**Background:**

Peripheral intravenous catheters (PIVC) are among the most common medical intervention for hospitalized patients. While PIVC can provide life-saving treatments, PIVC are also a major source of complications such as infiltration, occlusion, leaking, pain, dislodgement, and bacteremia, particularly due to *Staphylococcus aureus*.

Both the Infusion Nurses Society (INS) and the Centers for Disease Control and Prevention state that PIVC that are no longer in use should be removed, with INS specifically stating that a catheter that has not used for more than 24 hours should be removed. Despite the guidance to remove PIVC that are unused, prevalence studies demonstrate that approximately 14% of all PIVC have not been used in the prior 24 hours. These “idle PIVC” contribute to avoidable morbidity and mortality.

The goal of this study is to assess the opportunity for harm reduction at our institution by evaluating the prevalence of idle PIVC and the incidence of *S. aureus* bacteremia attributed to PIVC.

**Figure 1.**
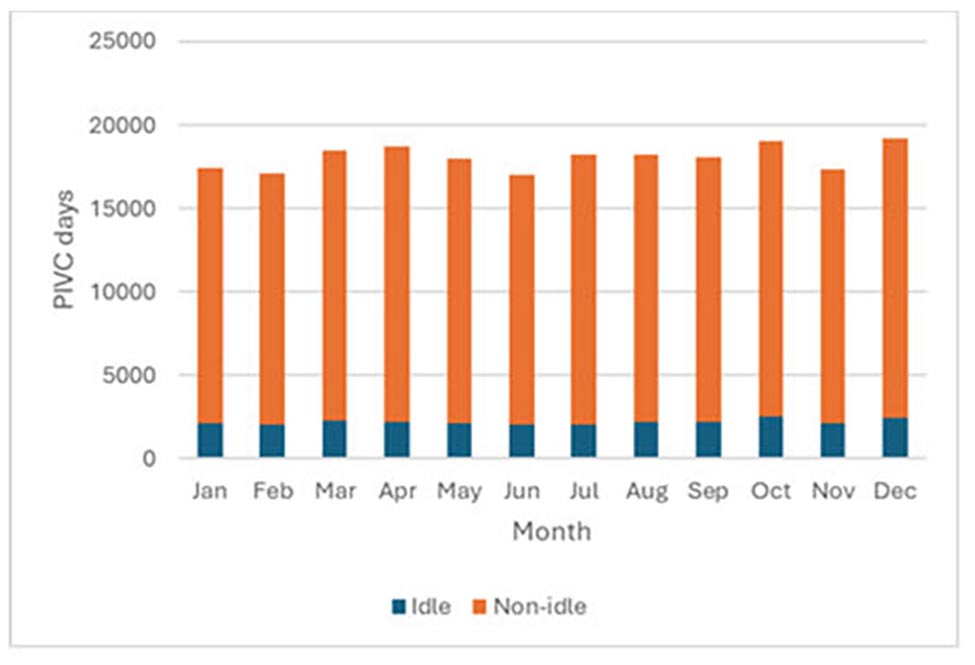
Peripheral intravenous catheter days in hospitalized patients, 2024

**Methods:**

This is a retrospective cohort study at a 500-bed academic safety net hospital in Denver, CO. The study dates are 1/1/24 through 12/31/24. All hospitalized patients were eligible for inclusion. PIVC were considered idle if they had not been used for at least 24 hours. Hospital onset *S. aureus* bacteremia was defined as those presenting on hospital day 4 or greater, with the day of admission being hospital day 1. A hospital-onset *S. aureus* bacteremia case was considered secondary to PVC if there was erythema, induration, or purulent drainage at the site of a PIVC that was currently in place or had been removed in the prior 7 days.

**Results:**

During the study period, there was a mean of 18,071 total PIVC days per month. The proportion of idle PIVC days ranged from 11.3% to 13.2% with a mean of 12.2% (Figure 1).

There were 7 cases of hospital onset *S. aureus* bacteremia (5 MSSA, 2 MRSA) attributed to PIVC during the study period. The rate of PIVC-related *S. aureus* bacteremia was 0.4 cases per 1000 PIVC days.

**Conclusion:**

Idle PIVC are prevalent in our institution. There is substantial opportunity to decrease the prevalence of idle PIVC. A reduction in idle PIVC could lead to fewer hospital onset *S. aureus* bacteremia cases.

**Disclosures:**

All Authors: No reported disclosures

